# A Shapelet Transform-Based Method for Structural Damage Identification: A Case Study on a Wooden Truss Bridge

**DOI:** 10.3390/s26082323

**Published:** 2026-04-09

**Authors:** Ke Gan, Yingzhuo Ye, Fulin Nie, Ching Tai Ng, Liujie Chen

**Affiliations:** 1 Guangzhou Testing Center of Construction Quality and Safety Co., Ltd., Guangzhou 510440, China; 2 School of Civil Engineering and Transportation, Guangzhou University, Guangzhou 510006, China; 3 School of Civil Engineering and Construction Management, Adelaide University, Adelaide, SA 5005, Australia

**Keywords:** shapelet transform, structural damage identification, structural health monitoring, random forest, feature extraction, timber truss bridge

## Abstract

The impact of environmental disturbances and sensor deployment variations on damage identification represents a critical bottleneck that constrains the practical effectiveness of structural health monitoring. Existing methods addressing these challenges often suffer from poor interpretability due to information loss during feature extraction or exhibit insufficient sensitivity in identifying early-stage minor damage. This paper proposes a damage identification method based on the Shapelet Transform and Random Forest classifier, which extracts highly interpretable local shape features from vibration response signals to achieve robust identification of structural state changes. The study utilizes measured random vibration response data from a timber truss bridge. The dataset comprises four reference states collected on different dates and five damage states simulated by additional masses ranging from +23.5 g to +193.7 g, with sensors deployed in both vertical and horizontal directions. The Shapelet Transform selects local subsequences with high information gain from the original time series as features, which are subsequently classified using the Random Forest algorithm. The experimental design systematically investigates the influence of different damage severities, sensor locations, and environmental variations on method performance. The results demonstrate that with a Shapelet extraction time of 10 min, the method achieves 100% identification accuracy across multiple operating conditions comprehensively considering environmental variations, sensor location differences, and varying damage severities. When the extraction time is reduced to 5 min, 3 min, and 1 min, the average accuracies are 93.98%, 89.51%, and 58.48%, respectively. The method effectively identifies the minimum simulated damage (+23.5 g), which represents only 0.07% of the total structural mass, while maintaining stable performance under varying sensor locations and environmental conditions. Compared to traditional methods based on global frequency-domain features or statistical characteristics, the proposed method extracts physically meaningful local Shapelet features, offering significant advantages in interpretability. In contrast to deep learning approaches, this method demonstrates greater robustness under limited sample conditions. This study confirms that the combined framework of the Shapelet Transform and Random Forest can effectively address multiple real-world challenges in structural health monitoring, delivering high accuracy, strong robustness, and excellent interpretability, thereby providing a novel approach for developing practical real-time damage identification systems.

## 1. Introduction

The safe operation and maintenance of civil infrastructure, particularly the sustainability and service life extension of historic or aging timber truss bridges, represents a critical challenge in contemporary engineering [1]. Timber structures are susceptible to damage such as localized decay, bolt loosening, and micro-cracks due to material aging, environmental erosion, and long-term loading. Failure to identify such damage in a timely manner may lead to severe consequences [2]. Structural health monitoring provides an essential means for real-time condition assessment and early warning of structures. However, SHM faces fundamental challenges in practical applications [3,4,5,6,7,8]: environmental and operational variations can induce significant alterations in vibration response signals, which often mask the subtle characteristic changes caused by early-stage, minor damage. Moreover, inconsistencies in sensor type, location, and density during actual deployment further complicate the reliable extraction of damage-sensitive features from data. Therefore, developing a damage identification method that is robust to environmental disturbances and sensor location variations is a bottleneck issue for enhancing the practical effectiveness of SHM.

Traditional vibration-based damage identification methods have been widely applied in SHM. Based on their feature extraction and modeling strategies, these methods can be broadly categorized into three types. Modal parameter-based methods identify damage by analyzing changes in structural frequencies, mode shapes, or damping. While these methods offer clear physical meaning, they often exhibit limited sensitivity to early-stage minor damage and are susceptible to interference from global factors such as ambient temperature variations. Damage induces changes in the physical parameters of a structure, leading to alterations in modal parameters including frequency, flexibility, stiffness, and mode shapes. Consequently, modal parameter-based approaches have been extensively explored for damage identification. Yan et al. [9] provided a comprehensive review of vibration-based structural damage identification methods, comparing the advantages and limitations of various techniques, including those based on frequency, flexibility, mode shapes, and modal strain energy. Structural damage typically reduces stiffness, resulting in changes in natural frequencies. Salawu [10] presented a detailed review of frequency-based damage identification methods. Pandey et al. [11] proposed a flexibility-based method based on the principle that structural flexibility changes upon damage occurrence. West et al. [12] introduced a mode shape-based method by comparing mode shapes before and after damage.

Signal processing and statistical feature-based methods, such as wavelet transform, principal component analysis, and autoregressive models, aim to extract statistical quantities or transform-domain features that reflect changes in structural state. However, these methods often suffer from information loss during feature construction, which compromises the interpretability of the results. Xin et al. [13] proposed a structural damage identification method combining a Swin Transformer and continuous wavelet transform. This approach first converts raw structural vibration data into time-frequency images using continuous wavelet transform to capture damage characteristics information, then employs Swin Transformer for hierarchical learning on these two-dimensional images to achieve efficient damage identification. Nouri et al. [14] successfully detected damage in timber bridges by integrating Fourier decomposition, time series modeling, and machine learning methods. Oliver et al. [15] proposed a wavelet transform-based damage identification method for laminated composite beams using modal and strain data, achieving high-quality damage detection. Liu et al. [16] developed a damage identification method based on extended Kalman filtering and response reconstruction, utilizing extended Kalman filters for signal filtering analysis and orthogonal matching pursuit algorithms for response reconstruction, significantly improving damage localization accuracy.

Machine learning-based methods employ various classifiers for pattern recognition on extracted features. Numerous recent studies have explored machine learning applications in SHM. For instance, Bayane et al. [17] applied unsupervised anomaly detection algorithms to bridge strain and acceleration data, constructing multiple feature matrices and evaluating five detection algorithms. Ren et al. [18] utilized support vector machines to identify damaged cables from strain responses of cable-stayed bridges, proposing a two-step method for damage localization and severity estimation. Ghiasi et al. [19] employed K-nearest neighbors classifiers to detect section loss caused by corrosion, achieving significantly improved detection accuracy. Soleimani et al. [20] used random forests to assess the importance of modeling parameters in seismic demand estimation. Flah et al. [21] provided a systematic review of machine learning algorithms in civil structural health monitoring, confirming their effectiveness across various structure types including bridges, buildings, and dams. Lim et al. [22] applied extreme gradient boosting to evaluate damage conditions of different bridge types using data from a bridge management system. Chang et al. [23] proposed stochastic deterioration models based on Markov chains and classification trees to address transition probability matrix estimation under limited data conditions. Sun et al. [24] developed a two-stage detection method combining Bayesian fusion and rough set theory, achieving efficient localization and assessment of multiple damages in bridges.

Despite the significant progress made by these methods, they often exhibit limitations when confronting complex environmental disturbances: some are overly sensitive to environmental variations, leading to elevated false alarm rates; others require extensive historical data covering diverse environmental conditions for modeling, incurring high costs. Their sensitivity is particularly limited when identifying early-stage, minor damage. Furthermore, many methods rely on specific, consistent sensor deployment schemes during feature extraction, and their identification performance and generalization capability may significantly deteriorate when the sensor network changes.

In response to these challenges, recent research has advanced toward more sophisticated and practical directions, particularly in handling real-world data and improving model reliability and interpretability. For instance, interpretable surrogate modeling techniques have emerged as a research focus in engineering failure analysis. These approaches aim to construct transparent models that not only achieve predictive accuracy but also reveal the physical logic between input features and structural responses, providing credible evidence for damage-related decisions [25]. Furthermore, data-driven frameworks integrating multi-source authentic data are gaining traction. Such frameworks, by incorporating diverse data types and sources, can more comprehensively and robustly characterize the performance evolution of complex structures under realistic operational conditions [26,27]. At the algorithmic level, deep learning methods combined with Bayesian optimization offer new solutions for hyperparameter tuning, small-sample learning, and uncertainty quantification, significantly enhancing the generalization capability and efficiency of models in complex structural damage identification tasks [28]. These cutting-edge explorations collectively point toward several key characteristics for future SHM methodologies: strong interpretability, multi-source data fusion capability, and robustness to real-world complexity and uncertainty.

Exploring novel approaches that directly mine discriminative features from raw dynamic response time series, while remaining relatively robust to environmental variations and sensor configuration changes, has become crucial for addressing current bottlenecks. Time series analysis techniques offer potential in this regard, as their core lies in discovering local morphological patterns from data that can characterize essential differences in structural states, rather than relying on indirect inferences from global statistics or model parameters. The Shapelet Transform represents a time series feature extraction method with such potential [29]. Its core concept involves identifying the “most discriminative subsequences” within time series, termed Shapelets—continuous subsequences that represent local shape characteristics of a particular class while distinctly contrasting with other classes. By computing distances between original sequences and a set of selected Shapelets, the Shapelet Transform transforms raw data into a discriminative feature space based on “shape similarity.” The key advantages of this method are twofold: the extracted features are themselves segments of the original signal, possessing intuitive physical or geometric meaning, thereby significantly enhancing model interpretability; simultaneously, it directly captures local morphological differences and is relatively insensitive to global amplitude variations potentially caused by environmental factors, aligning closely with the physical nature of damage-induced local dynamic characteristic changes emphasized in SHM [30]. Leveraging these advantages, Shapelet-based algorithms have been widely applied in various domains, including thunderstorm identification [31], earthquake and wind-wave prediction [32], sensor anomaly detection [33], motion capture [34,35], and medical diagnosis [36,37], demonstrating their effectiveness in extracting robust local features from noisy data.

However, introducing the Shapelet Transform into the civil engineering SHM domain, particularly for timber structures with more complex materials and configurations, presents several critical challenges requiring investigation. First, the material nonlinearity, connection complexity, and environmental sensitivity of timber structures result in more intricate vibration response characteristics compared to concrete or steel structures [38,39], necessitating thorough validation of the Shapelet Transform applicability and effectiveness in such scenarios. Second, existing studies have largely failed to systematically evaluate the comprehensive robustness of Shapelet Transform methods under multiple real-world challenges, including environmental disturbances, minor damage, and variations in sensor type and location [40], although some research has begun addressing environmental robustness issues in timber structure damage identification [41]. Third, the configuration of key parameters in Shapelet Transform methods significantly impacts final identification accuracy. However, systematic investigations into parameter effects and selection criteria tailored to civil engineering SHM scenarios remain lacking. While parameter studies in general time series classification [42] have demonstrated the importance of parameter selection, whether their conclusions can be directly transferred to civil SHM contexts requires further verification.

Addressing these challenges, this paper proposes a damage identification framework combining the Shapelet Transform with a random forest classifier, aiming to provide a novel approach with high interpretability and robustness for anomaly detection in SHM data. As a representation technique based entirely on local shapes of time series, the Shapelet Transform captures distinctive local waveform patterns in sensor anomaly data, while the random forest classifier utilizes these morphological features to effectively identify and classify different anomaly patterns within large SHM databases. Based on actual measurement random vibration response data from a timber truss bridge, this study systematically investigates the recognition performance of the proposed method under various conditions, including different damage severities, sensor locations, and environmental variations. The experimental results demonstrate that the proposed method not only achieves high-precision damage identification but also exhibits sensitivity to early-stage minor damage and robustness to sensor location and environmental disturbances, offering a new technical pathway for developing interpretable and adaptable structural health monitoring methodologies.

The structure of this paper is as follows. Section 2 provides an overview of the fundamental principles and algorithmic process of the Shapelet Transform. Section 3 introduces the timber truss bridge SHM dataset employed in this study, systematically presenting damage identification results under different damage severities, sensor locations, and environmental influences, as well as comparing the damage identification performance of different classifiers. Section 4 discusses the influence of different Shapelet extraction times on identification accuracy, analyzes the reasons for performance differences between random forest, KNN, and SVM under various operating conditions, and conducts in-depth analysis of morphological characteristics of selected Shapelet waveforms, exploring their physical correlation with structural damage severity. Section 5 summarizes the main conclusions of this study, identifies the advantages and limitations of the proposed method, and presents prospects for its future application in real-time monitoring.

## 2. Proposed Method

### 2.1. Shapelets

**Figure 1:**
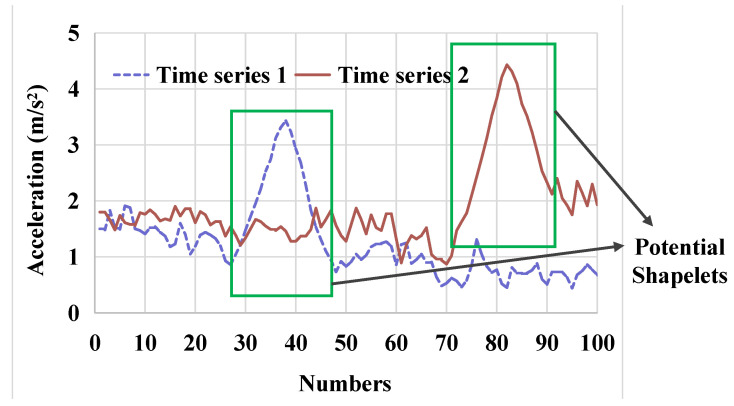
Time series and Shapelets.

A subsequence with a clearly distinct shape feature that appears locally in time series collected for an event is called a Shapelet, as shown in Figure 1. It can be seen from Figure 1 that the Shapelet sustains a short time and is distinguished from the rest of the time series. Generally, the analysis process of the classification method based on the global time series is a black box. The Shapelet provides an easily understood way based on local shape features of time series, which is similar to visual object detection. These local feature shapes are phase and amplitude-independent, which is conducive to further classification and recognition tasks.

### 2.2. Shapelet Transform

Computing similarity between Shapelets and time series as a discriminant feature to solve time series classification problems has attracted significant research attention in recent years. The classification algorithms based on Shapelets were originally proposed by Ye et al. [43], which embed Shapelets’ discovery process into decision trees and use Information Gain (IG) to evaluate Shapelets’ quality. However, this classification process is complicated and cannot be applied in most cases. Lines et al. [29] proposed the Shapelet Transform method to decouple the discovery process of Shapelets from the classifier. The algorithm first selects the best *k* Shapelets from the data set. It then converts the distance from each time series to these Shapelets into *k* attributes, thereby projecting the original data set onto a new feature space. This transformation improves classification accuracy while retaining the interpretability of the Shapelets. The resulting feature space allows for the integration of various classifiers, which can be selected based on the specific application requirements. Based on the obtained Shapelets, the time series data can be transformed from the time domain to the Shapelet domain through the Shapelet Transform algorithm, and combined with machine learning classification algorithms to effectively classify and identify the data. The three main steps of Shapelet Transform are described as follows:

#### 2.2.1. Step 1: Selection of Shapelets

**Figure 2:**
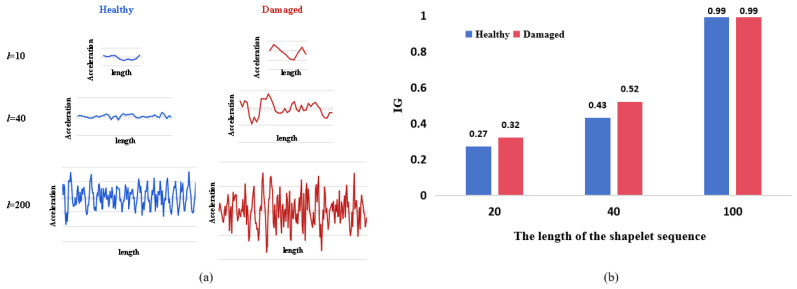
Candidate Shapelets: (**a**) is Candidate Shapelet waveforms of different lengths; (**b**) is Information gain (IG) values of the corresponding candidate Shapelets.

Firstly, the Shapelet candidates of the time series dataset containing all subsequences are generated by brute force search. In the wooden truss bridge example of this paper, the length of the training and testing set $TS=\{TS_{1,\;}TS_{2},\ldots ,\;TS_{N}\}$ in each time series is 8192. Taking *TS*_1_ as an example, the union of all candidate Shapelets in *TS*_1_ can be represented as follows:

(1) $$W_{1}=\{\omega_{3},\;\omega_{4},\ldots ,\;\omega_{8191},\;\omega_{8192}\}$$

where $\omega_{3}$ represents a total of 8190 candidate Shapelets with the minimum length (*l* = 3), and $\omega_{8192}$ represents the maximum length (*l* = 8192) of the candidate Shapelets. The candidate Shapelets for length 10, 40, 200 in this paper are shown in Figure 2.

**Figure 3:**
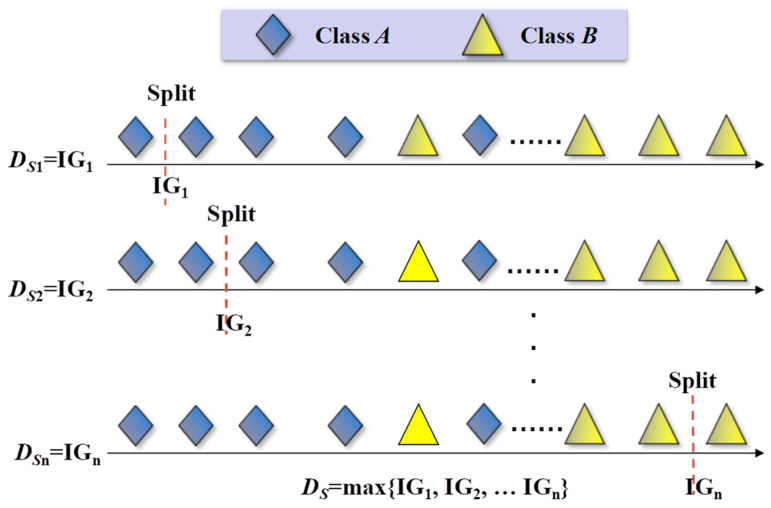
Segmentation rule based on IG value of Euclidean distance.

Then, the Shapelet candidates should be evaluated for quality through IG. IG is an important metric for feature selection, which is used to remove irrelevant or redundant features and reduce the dimensionality of the data. IG measures the reduction in entropy achieved by splitting a dataset based on a particular attribute [32]. In this study, IG is used to evaluate the quality of candidate Shapelets. When a dataset *S* contains two different classes of *A* and *B*, the entropy of *S* is given by:

(2) E(S)=−p(A)log(p(A))−p(B)log(p(B))

where *p*(*A*) is the proportion of class *A* in *S*, and *p*(*B*) is the proportion of class *B* in *S*. The dataset *S* is divided into two datasets *S*_A and *S*_*B* by segmentation rules. In this paper, the segmentation rule is the Euclidean distance between the candidate Shapelets and each time series. The segmentation point is set at the midpoint of two adjacent distances as shown in Figure 3. IG is calculated by the following formula.

(3) $$\mathrm{IG}=E(S)-(\frac{\left|S_{A}\right|}{\left|S\right|}E(S_{A})+\frac{\left|S_{B}\right|}{\left|S\right|}E(S_{B}))$$

where 0 ≤ IG ≤ 1. To retain useful features to facilitate further classification, the minimum IG threshold must be set appropriately for each calculation example. If the IG value of a candidate Shapelet is less than the set threshold, the Shapelet is discarded; otherwise, it is retained.

#### 2.2.2. Step 2: Transformation to Shapelet Domain

Shapelet Transform is performed after high-quality Shapelets are selected that contain classification information. Then, the time series data are transformed to the Shapelet domain. The value of the converted Shapelet domain is derived from calculating the similarity by Euclidean distance between the time series and the corresponding Shapelets in this paper. The smaller the Euclidean distance, the greater the similarity between the candidate Shapelet and the test time series. Therefore, when the candidate Shapelets belong to the same category as the test time series, they have a smaller distance that indicates a greater similarity. Whereas, when the candidate Shapelets and the test time series do not belong to same category, they have a larger distance indicating a smaller similarity. The squared Euclidean distance between a candidate Shapelet *X* of length *l* and a test time series subsequence *Y* of the same length *l* is defined as:

(4) $$\mathrm{dis}(X,Y)={\left[{\sum_{i=1}^{l}\left(X_{i}-Y_{i}\right)}^{2}\right]}^{0.5}$$

**Figure 4:**
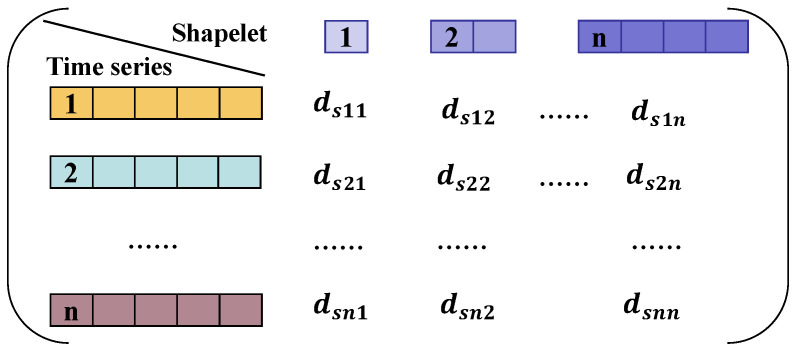
Shapelet domain matrix.

A list containing *n* distances is constructed and denoted as *DS* by computing the Euclidean distance between candidate Shapelets and all test time series in *TS. DS* consists of the Euclidean distance value and the category label for all the candidate Shapelets. Each of the element features *d_s_*_nn_ in the Shapelet domain matrix shown in Figure 4 represent a distance between a Shapelet and a test time series, where D_S11_ represents the distance between Shapelet 1 and time series 1, with the rest defined analogously.

#### 2.2.3. Step 3: Random Forest Classifier

The classification issue of time series is transformed into a general classification problem after the Shapelet transform, which can be combined with different classification methods. Ye et al. [30] initially developed a decision tree classifier algorithm based on Shapelets. This algorithm evaluates events based on Shapelets and the corresponding distance thresholds may lead to underfitting or overfitting. To avoid this problem, researchers have developed more advanced classifiers [30,44]. In this paper, the random forest classifier is adopted to further classify and identify structural vibration response based on Shapelets. The random forest classifier contains multiple decision trees, and each node of the decision tree represents the condition of a time series feature. This avoids the influence of outliers and overfitting caused by a single decision tree. In this paper, based on the local Shapelet features of the structural vibration response in reference and damage conditions, the Shapelet domain training dataset is trained with the random forest classifier, and then the trained classifier is used for the test dataset.

### 2.3. The Process of Structural Damage Identification Based on Shapelet Transform

The basic ideas and procedure of the proposed structural damage identification method combining Shapelet Transform and machine learning algorithms in this paper are described as follows: (1) constructing a dataset of structural vibration response and labeling them by categories; (2) selecting effective Shapelets from the dataset through the Shapelet Transform algorithm to convert them into the Shapelet domain as a distance matrix; and (3) classifying and identifying the dataset using machine learning by a random forest classifier.

## 3. Damage Identification of a Wooden Truss Bridge

### 3.1. Dataset and Test Setup

**Figure 5:**
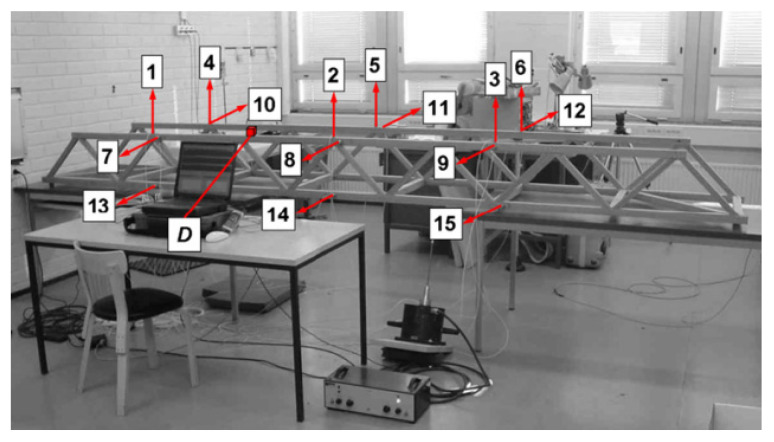
The wooden truss bridge: 1~15 is the sensor number, and point *D* is the damage position [42].

The dataset utilized in this study was obtained from an experimental wooden truss bridge structure [45], illustrated in Figure 5. Broadband random excitation was applied to the structure using an electrodynamic shaker mounted on the bridge, simulating ambient vibration and exciting multiple structural modes. Accelerometers (Kistler 8712A5M1) were installed at 15 locations to capture the dynamic response under steady-state random vibration. The signals were sampled at 256 Hz over a duration of 32 s, resulting in 8192 data points per measurement. To simulate the inevitable environmental and operational variations encountered in real-world structural health monitoring, reference condition data were collected on multiple different dates: (1) damage condition data were collected on 28th May, and different masses (23.5 g, 47.0 g, 70.5 g, 123.2 g and 193.7 g) were attached on the top flange at point D to simulate damage; (2) reference condition data were collected on 18th May, 25th May, 28th May and 29th May, respectively. The details of the data collection are shown in Table 1, where data types a to i refer to nine conditions of vibration responses in the training and testing datasets. The experiment was repeated ten times for each condition. Natural fluctuations in ambient temperature and humidity across these different dates serve as the primary source of environmental variations. Additionally, during the data measurement period, a fan was operating in the laboratory, and the resulting airflow disturbances may have had minor effects on the ambient vibration excitation of the structure, further increasing the complexity of the environmental conditions. Meanwhile, to simulate sensor faults characterized by reduced measurement accuracy, random noise with a standard deviation of σ = 0.001 was added to the data from Sensor 3. These settings collectively constitute a comprehensive testing scenario incorporating multiple environmental and operational variation factors, which is used to evaluate the robustness of the proposed algorithm for damage identification when confronted with real-world environmental disturbances.

The training dataset and testing dataset were divided in a ratio of 7:3 to meet the requirements of the algorithm proposed in this paper. The parameters of the proposed algorithm were then set according to Section 2. The extraction times for Shapelets were set to 1 min, 3 min, 5 min, and 10 min, respectively, to balance the efficiency of extraction and ensure that all high-quality Shapelets can be selected. It can be predicted that a longer extraction time is more effective to ensure all selected Shapelets have high IG values. The calculation was performed in Python 3.8 using a computer with an Intel(R) Xeon(R) W-2245 CPU @ 3.90 GHz.

### 3.2. Damage Identification Results Based on Shapelet Transform

In this study, the data from the wooden truss bridge was used to verify the proposed damage identification method based on Shapelet Transform combined with a random forest classifier. The influence of damage degree, sensor location, and environmental factors were considered. A total of 15 tests are divided into conditions I, II and III, as shown in Table 2.

The results of the damage identification by different Shapelet extraction times, such as 1 min, 3 min, 5 min and 10 min, are shown in Table 3.

According to Table 3, the damage identification method based on Shapelet Transform combined with random forest classifier achieved satisfactory results for the data obtained from the wooden truss bridge. When the Shapelet extraction time was set to 10 min, the average accuracy of the three conditions are all 100%, even with the influence of sensor position, varying environmental condition, and different degrees of damage. When the Shapelet extraction time was reduced to 1 min, the accuracy of conditions I, II and III is 58.33%, 65.45%, and 51.67%, respectively, with a total average accuracy of 58.48%. When the Shapelet extraction time was reduced to 3 min, the accuracy of conditions I, II and III is 88.89%, 91.67%, and 87.96%, respectively, with a total average accuracy of 89.51%. When the extraction time was set to 5 min, the accuracy of conditions I, II and III is 90.28%, 97.22%, and 94.44%, respectively, with a total average accuracy of 93.98%. Therefore, as the Shapelet extraction time increases, the accuracy of the proposed algorithm improves effectively.

**Figure 6:**
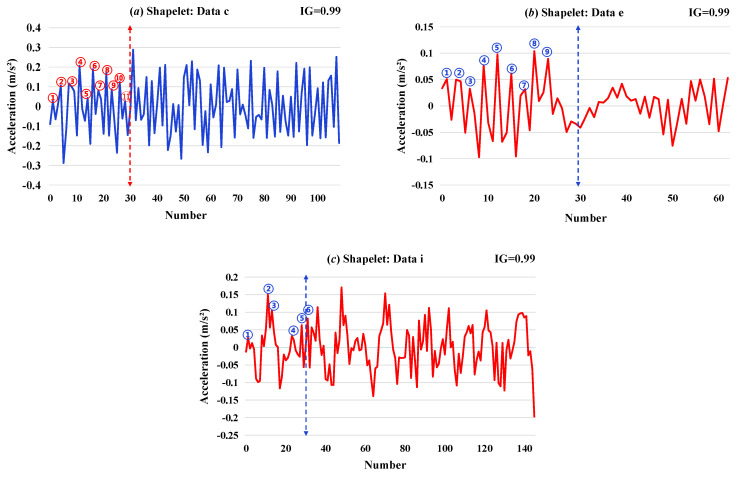
Shapelets selected in condition I: (**a**) data c; (**b**) data e; and (**c**) data i. (Each circle in the figure indicates one waveform peak.)

**Figure 7:**
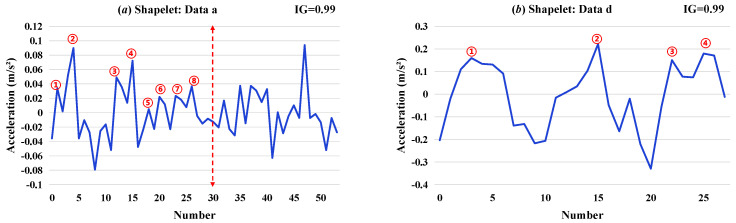

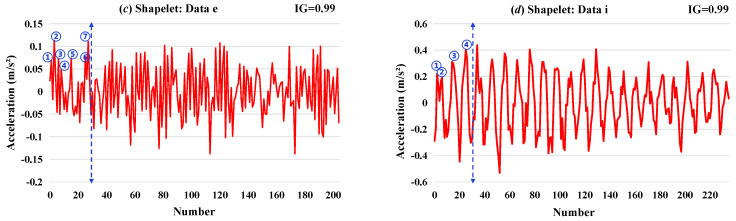
Shapelets selected in condition II: (**a**) data a; (**b**) data d; (**c**) data e; and (**d**) daata i. (Each circle in the figure indicates one waveform peak.)

**Figure 8:**
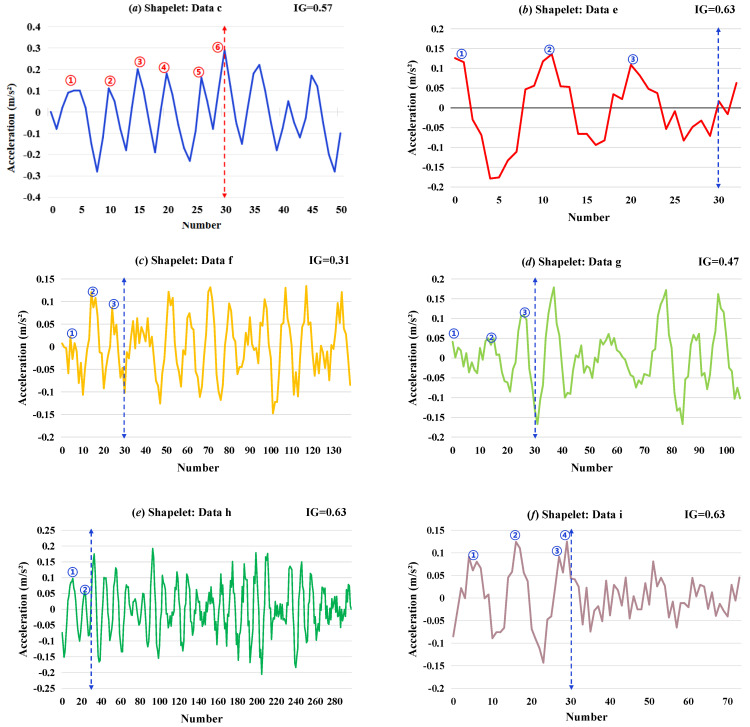
Shapelets selected in condition III: (**a**) data c; (**b**) data e; (**c**) data f; (**d**) data g; (**e**) data h; and (**f**) data i. (Each circle in the figure indicates one waveform peak.)

Then, the recognition results under different conditions with sufficient Shapelet extraction time (10 min) were analyzed and are shown in Figure 6, Figure 7 and Figure 8.

From Figure 6, Figure 7 and Figure 8, it shows that the IG values of the Shapelets are all above 0.05, indicating that the selected Shapelets have high classification quality. In that case, the selected Shapelets can effectively combine with machine learning algorithms as a tool for data classification and identification. As shown in Figure 6, in the condition I of (1)–(8), sensors 1 and 7 are arranged in vertical position, while sensors 6 and 12 are in horizontal position, where “e” represents the minimum damage category and “i” represents the maximum damage category. From the selected Shapelets in Figure 6, it shows that the local features of Shapelets under the reference condition are significantly different from those under damaged conditions. In the category of reference conditions, the curve of the selected Shapelets is mainly characterized by random steady fluctuation. The category of small damage is mainly characterized by single abnormal fluctuations, and the category of large damage is mainly characterized by periodic abnormal fluctuations. Moreover, the selected local feature from Shapelets can ensure that the identification of small damage is independent to sensor location.

In the condition II of (9)–(14), the influences of both the sensor position and the environmental conditions were considered. From the local feature from the selected Shapelets in Figure 7, it shows that the selection strategy for Shapelets is significantly different from that in condition I. To effectively detect the damage condition, similar local features of Shapelets were selected under different damage conditions, while Shapelets that highlight the impact of environmental factors were selected under the reference condition.

In condition III of (15), the influence of different degrees of damage on the identification is investigated. From the selected Shapelets from Figure 8, it shows that under the reference condition, the selected Shapelets exhibit stable random fluctuations, while under different degrees of damage, the selected Shapelets exhibit abnormal fluctuations of different periods.

To further investigate the physical interpretability of Shapelet features, this study conducts morphological observations on representative Shapelet waveforms selected by the algorithm, as shown in Figure 6, Figure 7 and Figure 8. By counting the number of peaks within the first 30 data points of each Shapelet, the following patterns are identified:

First, under the same acquisition date, significant differences in waveform morphology are observed between the healthy state and damaged states. As illustrated in Figure 6, the Shapelet corresponding to the healthy state (Figure 6a) contains 11 peaks within the first 30 data points, while those corresponding to the minimum damage state (Figure 6b) and maximum damage state (Figure 6c) contain only 9 and 6 peaks, respectively. This decreasing trend indicates that as the additional mass increases, the oscillation frequency of local vibration waveforms gradually decreases.

Second, Figure 8 further validates this pattern. Among the six different states collected on 28th May, the healthy state (Figure 8a) exhibits a peak count significantly exceeding 4, whereas the peak counts of the five damaged states (Figure 8b–f) are concentrated between 3 and 4. Notably, the minimum damage state contains 4 peaks, while all other more severe damage states contain only 3 peaks, suggesting a positive correlation between the reduction in peak count and the escalation of damage severity.

Furthermore, Figure 7 demonstrates the influence of environmental factors on waveform morphology. The healthy state on 28th May (Figure 7a) contains 8 peaks, while the healthy state on 29th May (Figure 7b) contains only 4 peaks, indicating that environmental conditions on different dates also affect the local waveform characteristics of vibration responses. However, even under environmental variations, the peak counts of damaged states (Figure 7c,d) still exhibit clear differences from those of healthy states, providing a morphological explanation for the algorithm’s ability to maintain high identification accuracy under environmental disturbances.

These observations indicate that the local waveform features extracted by the Shapelet algorithm possess not only mathematical discriminative power, but also morphological information closely related to changes in the physical state of the structure. Variations in peak count can be regarded as intuitionistic manifestations in the time-domain waveform of damage-induced alterations in local dynamic characteristics, thereby providing compelling visual evidence for the interpretability of the proposed method.

### 3.3. Performance Comparison of Different Classifiers

To empirically validate the rationality of selecting the Random Forest classifier within the proposed damage identification framework, this study conducts a comparative analysis with two alternative mainstream classifiers: K-Nearest Neighbors and Support Vector Machine. The comparative experiments are performed under a Shapelet extraction time of 5 min, with the rationale for this parameter selection and classifier choices elaborated as follows.

The extraction time of 5 min represents a moderate configuration among the four tested time windows. As demonstrated in Section 3.2, while the 10 min extraction achieves perfect classification, the 5 min setting maintains high average accuracy (93.98%) while significantly reducing computational cost compared to the 10 min configuration. This time point therefore provides a balanced scenario for classifier evaluation—it offers sufficiently discriminative Shapelet features while avoiding the ceiling effect observed at 10 min. Evaluating classifier performance under this moderate configuration allows for meaningful differentiation of their respective capabilities in utilizing the same Shapelet feature space.

Selection of Comparative Classifiers—KNN and SVM are selected as benchmark classifiers for the following reasons:

First, KNN represents a fundamental instance-based learning approach that classifies samples based on their proximity in the feature space. Its simplicity and lack of training phase make it an intuitive baseline for evaluating the inherent separability of the Shapelet feature space. The performance of KNN directly reflects the quality of distance metrics in the transformed feature domain, providing insights into whether Shapelet features naturally cluster according to damage categories.

Second, SVM represents a powerful margin-based classification paradigm with well-established theoretical foundations. Its ability to construct optimal separating hyperplanes in high-dimensional spaces through kernel functions makes it particularly relevant for comparing against Random Forest’s ensemble approach. The performance comparison reveals whether the Shapelet feature space benefits more from margin maximization strategies (SVM) or ensemble-based decision boundaries (Random Forest).

Third, both classifiers exhibit distinct characteristics relevant to SHM applications:

(1) Interpretability trade-offs: KNN offers limited interpretability beyond local neighbor relationships, while SVM provides decision boundaries that are difficult to map back to physical waveform patterns. Comparing these against Random Forest’s feature importance rankings helps validate the interpretability advantages claimed for the proposed framework.

(2) Robustness to high-dimensional features: KNN suffers from the curse of dimensionality, while SVM requires careful parameter tuning. Their performance relative to Random Forest, which naturally handles high-dimensional features, provides insights into the characteristics of the Shapelet-transformed feature space.

(3) Sample efficiency: Both KNN and SVM have well-documented behaviors under limited sample conditions, making them suitable benchmarks for evaluating Random Forest’s ensemble-based robustness with the current dataset size.

**Figure 9:**
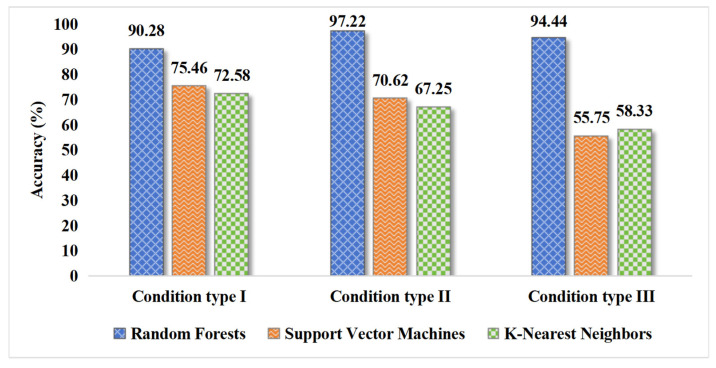
Recognition accuracies of Random Forest, KNN, and SVM classifiers under different operating conditions with a 5 min Shapelet extraction time.

All three classifiers are evaluated using identical training and test set partitions (7:3 ratio) within the same Shapelet feature space generated with a 5 min extraction time. The recognition accuracies under Conditions I, II, and III are presented in Figure 9 and analyzed in detail below.

As shown in Figure 9, under the same Shapelet feature space, Random Forest achieves significantly higher recognition accuracies than KNN and SVM across all three conditions. Under Condition I, Random Forest achieves an accuracy of 90.28%, outperforming KNN (72.58%) and SVM (75.46%) by 17.70 and 14.82 percentage points, respectively. Under Condition II, Random Forest attains an accuracy of 97.22%, while KNN and SVM achieve only 67.25% and 70.62%, respectively, further widening the performance gap. Under Condition III, Random Forest achieves an accuracy of 94.44%, whereas KNN and SVM drop to 58.33% and 55.75%, respectively, indicating that the latter two classifiers struggle in fine-grained multi-class classification tasks.

## 4. Discussion

### 4.1. Overall Identification Performance Analysis

This study achieves satisfactory identification results in the timber truss bridge damage identification task by combining the Shapelet Transform with the Random Forest classifier. As shown in Table 3, the Shapelet extraction time has a significant impact on identification accuracy: when the extraction time is 1 min, the average accuracy across the three operating conditions is 58.48%; at 3 min, the average accuracy increases to 89.51%; when extended to 5 min, the average accuracy improves to 93.98%; and when the extraction time reaches 10 min, the identification accuracy achieves 100% across all conditions. This result indicates that sufficient Shapelet extraction time is crucial for ensuring feature quality—longer extraction times allow the algorithm to screen local shape features with higher information gain and stronger discriminative power from the vast number of candidate subsequences, thereby constructing a more discriminative feature space.

Examining the identification results across different operating conditions, the proposed method demonstrates stable and high performance in Conditions I, II, and III.

In Condition I, the algorithm is required to identify data from different sensor locations. The average accuracy reaches 90.28% with a 5 min extraction time and achieves 100% at 10 min. This indicates that the local shape features extracted by the Shapelet Transform are insensitive to sensor deployment direction—whether sensors are positioned vertically or horizontally, the local waveform patterns excited by the same damage state exhibit similarity, and the algorithm can capture these common features across different locations.

In Condition II, the test data comprise a mixture of reference states and damage states collected on different dates. The accuracy reaches 97.22% with a 5 min extraction time and also achieves 100% at 10 min. This result validates the excellent robustness of the proposed method against environmental factors. Although differences in temperature and humidity across different dates may cause shifts in the overall dynamic characteristics of the structure, the Shapelet algorithm, by focusing on local waveform morphology, can effectively distinguish global changes induced by environmental factors from local anomalies caused by damage, thereby maintaining stable identification performance.

In Condition III, the algorithm is required to simultaneously distinguish among six categories, including the healthy state and five different damage severities, representing a typical fine-grained multi-class classification task. The accuracy reaches 94.44% with a 5 min extraction time and also achieves 100% at 10 min. Of particular note, the algorithm successfully distinguishes the minimum simulated damage, which represents only 0.07% of the total structural mass, from both the healthy state and other damage severities. This demonstrates the method’s exceptional sensitivity to early-stage minor damage—a sensitivity derived from the Shapelet Transform’s capability to finely capture local waveform details and extract subtle feature differences caused by minor mass variations from the noise background. Furthermore, the algorithm successfully differentiates among five distinct damage severities, demonstrating that the Shapelet feature space possesses the capability for the fine-grained quantification of damage severity beyond merely discriminating the presence or absence of damage.

The following Section 4.2 and Section 4.3 will provide in-depth analyses of the performance advantages of the proposed method from two dimensions: classifier selection and Shapelet waveform morphology, respectively.

### 4.2. Correlation Between Shapelet Waveform Morphology and Physical Damage

This section further explores the inherent relationship between the morphological characteristics of Shapelet waveforms and changes in the physical state of the structure. From the observations in Section 3.2, it can be found that under the same acquisition date, Shapelet waveforms corresponding to the healthy state exhibit the highest density of peak counts, while those corresponding to damaged states show significantly reduced peak counts, with this decreasing trend generally consistent with the escalation of damage severity. This phenomenon can be explained from the perspective of structural dynamics.

When the structure is in a healthy state, it possesses relatively high local stiffness and low damping, resulting in higher frequency oscillations in response to external excitation, which manifests as a denser distribution of peaks in the time-domain waveform. As damage is introduced, the local dynamic characteristics of the structure undergo changes: the increase in equivalent mass reduces local modal frequencies, while the decrease in stiffness also leads to frequency reduction, accompanied by potential increases in damping. The combined effect of these changes prolongs the oscillation period of the vibration response, correspondingly reducing the number of peaks. Therefore, the decreasing trend in peak count can be regarded as a quantitative characterization of escalating damage severity in the time-domain waveform.

It is noteworthy that the differences in peak counts of healthy states on different dates shown in Figure 7 reveal the significant influence of environmental factors on waveform morphology. Variations in environmental conditions such as temperature and humidity affect the elastic modulus of timber and the frictional characteristics of connection nodes, thereby causing overall shifts in structural dynamic properties. However, within the same date, the differences in peak counts between healthy and damaged states remain significant, indicating that the Shapelet algorithm can effectively distinguish global changes induced by environmental factors from local changes caused by damage—the former may lead to overall shifts in waveform morphology, while the latter are reflected in specific morphological alterations of local subsequences.

The findings hold important engineering implications. On one hand, intuitive morphological indicators such as peak count can serve as auxiliary criteria to help engineers understand why the algorithm identifies a particular signal segment as “damage.” On the other hand, this pattern also provides insights for developing more lightweight damage identification methods in the future—for instance, simple features such as peak density statistics of local waveforms could be used for preliminary screening, followed by precise classification using the Shapelet Transform.

In summary, the Shapelet Transform is not merely a data-driven feature extraction method; the local subsequences it selects inherently carry morphological information closely related to changes in the physical state of the structure. This “features-as-explanation” characteristic constitutes the core advantage of the proposed method compared to traditional “black-box” models.

### 4.3. Analysis of Classifier Performance Differences

The comparative results presented in Section 3.3 demonstrate that, based on the same Shapelet features, the recognition performance of different classifiers varies significantly. The reasons can be attributed to the following aspects:

First, the ensemble mechanism of Random Forest effectively enhances generalization capability. Random Forest constructs multiple decision trees through bootstrap sampling and integrates their voting results, which effectively reduces model variance and avoids overfitting to specific noise in the training data [46]. This characteristic is particularly prominent under Condition II—when the test data contains environmental fluctuations from different dates, the accuracies of KNN and SVM drop to 67.25% and 70.62%, respectively, while Random Forest maintains a high accuracy of 97.22%, demonstrating the robustness of its ensemble mechanism against environmental disturbances.

Second, the high-dimensional nature of the Shapelet feature space imposes different requirements on classifiers. The Shapelet Transform converts the original time series into vectors composed of distances to multiple Shapelets. This feature space is typically high-dimensional and exhibits complex nonlinear relationships among features. Random Forest, with its decision tree-based algorithmic structure, can naturally handle high-dimensional features and identify the most critical Shapelets for classification through its feature selection mechanism. In contrast, KNN is susceptible to the “curse of dimensionality” in high-dimensional spaces, leading to diminished discriminative power of distance metrics, which explains its accuracy of only 58.33% under Condition III. Although SVM can address nonlinear problems through kernel functions, its performance heavily depends on parameter tuning and struggles to achieve optimality with limited samples.

Third, Random Forest forms a synergy with the interpretability of Shapelet features. Random Forest can output feature importance rankings, helping to identify which Shapelets are most critical for damage identification. This characteristic complements the inherent interpretability advantage of the Shapelet Transform, enabling the entire methodological framework to not only achieve high accuracy but also provide intuitive physical evidence for engineering decisions. In contrast, neither KNN nor SVM can offer comparable interpretability support in this dimension.

In summary, the classifier comparison results empirically validate the rationality of selecting Random Forest in this study. It not only maximizes the utilization of discriminative information from Shapelet features but also demonstrates significant performance advantages under real-world challenges such as environmental disturbances, sensor variations, and fine-grained multi-class recognition tasks.

## 5. Conclusions

This study addresses core challenges in structural health monitoring, including environmental disturbances, sensor deployment variations, and the difficulty of identifying early-stage minor damage, by proposing a novel damage identification method that combines the Shapelet Transform with a Random Forest classifier. The proposed method mines highly discriminative local shape subsequences from original vibration response time series to construct an interpretable feature space, and leverages the ensemble mechanism of Random Forest to achieve robust classification. Based on a timber truss bridge experimental dataset, the effectiveness of the method is systematically validated under various conditions involving different damage severities, sensor locations, and environmental variations. The main conclusions are as follows.

First, as a highly interpretable local feature extraction method, the Shapelet Transform demonstrates good applicability in timber structure damage identification. Experimental results show that with a Shapelet extraction time of 10 min, the method achieves 100% identification accuracy across Condition I, Condition II, and Condition III. When the extraction time is reduced to 5 min, the average accuracy across the three conditions remains at 93.98%. Even with an extraction time of only 3 min, the average accuracy maintains 89.51%. These quantitative results reveal a positive correlation between Shapelet extraction time and identification accuracy—longer extraction times allow the algorithm to screen local features with higher information gain, thereby constructing a more discriminative feature space.

Second, the proposed method exhibits significant sensitivity to early-stage minor damage and demonstrates good robustness to sensor location variations and environmental changes. For the minimum simulated damage, which represents only 0.07% of the total structural mass, the method achieves effective identification with a 5 min extraction time. Under Condition I, the algorithm stably identifies data from both vertically and horizontally deployed sensors, indicating that Shapelet features are insensitive to sensor location. Under Condition II, facing environmental fluctuations such as temperature and humidity variations inherent in reference data collected on different dates, the method achieves accuracies of 97.22% and 100% with extraction times of 5 min and 10 min, respectively, demonstrating its ability to effectively distinguish global changes caused by environmental factors from local anomalies induced by damage. Condition III requires the algorithm to simultaneously distinguish among six categories, including a healthy state and five different damage severities, representing a typical fine-grained multi-class classification task. With a 5 min extraction time, the accuracy reaches 94.44%, and with 10 min, it achieves 100%. The algorithm not only accurately distinguishes the healthy state from various damage severities but also successfully differentiates among the five damage severities, demonstrating that the Shapelet feature space possesses the capability for fine-grained quantification of damage severity beyond merely discriminating the presence or absence of damage.

Third, the selection of the Random Forest classifier is validated through quantitative comparison. Within the same Shapelet feature space (5 min extraction time), Random Forest achieves significantly higher accuracies than K-Nearest Neighbors and Support Vector Machines across Conditions I, II, and III. This comparison indicates that the ensemble mechanism of Random Forest not only effectively utilizes the high-dimensional discriminative information of Shapelet features but also exhibits significant performance advantages under real-world challenges such as environmental disturbances, sensor variations, and fine-grained multi-class classification.

Fourth, Shapelet features possess clear physical interpretability, with their waveform morphology exhibiting inherent correlation with structural damage severity. Morphological observation of selected Shapelet waveforms reveals that under the same acquisition date and identical subsequence length, the healthy state exhibits a significantly higher number of peaks compared to both the minimum damage state and the maximum damage state. As damage severity increases progressively from minimum to maximum, the peak count shows a decreasing trend. This pattern indicates that the reduction in local stiffness and increase in mass caused by damage lead to prolonged oscillation periods and reduced peak density in vibration responses—physical changes that are precisely captured as distinguishable morphological features by the Shapelet algorithm. This “features-as-explanation” characteristic constitutes a core advantage of the proposed method compared to traditional “black-box” models.

Despite these achievements, this study has certain limitations. First, the experiment simulates damage by attaching mass blocks, which differs in physical mechanisms from real progressive damage such as timber decay and crack propagation. Second, the current Shapelet discovery strategy based on an exhaustive candidate search incurs high computational costs, potentially limiting its application in ultra-long time series or real-time scenarios. Future research will proceed in the following directions: (1) further validating the effectiveness of the proposed method on bridge monitoring data containing real timber damage; (2) exploring fast Shapelet discovery algorithms based on heuristic search or learning to reduce computational costs; (3) extending the method to other material structures such as steel and concrete to verify its generalization capability; (4) developing a real-time monitoring prototype system based on an “offline training, online lightweight inference” architecture to facilitate the translation of the method into engineering practice.

In summary, this study not only validates the effectiveness of the Shapelet Transform in structural damage identification but also provides a novel technical pathway for developing interpretable, robust, and online-capable intelligent identification algorithms. The proposed methodological framework exhibits clear application potential in scenarios such as historic timber bridge monitoring, the rapid assessment of bridges in remote areas, and the intelligent upgrading of existing monitoring systems.

## Figures and Tables

**Table 1:** Data collection list.

Data Type	Acquisition Date	Sensor Numbers	Data Description
a	18th May	1~15	Reference condition
b	25th May	1~15	Reference condition
c	28th May	1~15	Reference condition
d	29th May	1~15	Reference condition
e	28th May	1~15	Damaged condition (+23.5 g, Minimum damage)
f	28th May	1~15	Damaged condition (+47.0 g)
g	28th May	1~15	Damaged condition (+70.5 g)
h	28th May	1~15	Damaged condition (+123.2 g)
i	28th May	1~15	Damaged condition (+193.7 g, Maximum damage)

**Table 2:** Test conditions.

Test Number	Data Type	Sensor Number	Condition Description
(1)	c, i	1	I: Set up the tests to verify the influence of the algorithm on the recognition accuracy of large and small damage and the sensor position
(2)	c, e	1	
(3)	c, i	6	
(4)	c, e	6	
(5)	c, i	7	
(6)	c, e	7	
(7)	c, i	12	
(8)	c, e	12	
(9)	a, i	2	II: Set up the tests to verify the influence of environment and sensor position
(10)	a, e	3	
(11)	b, i	5	
(12)	b, e	8	
(13)	d, i	11	
(14)	d, e	13	
(15)	c, e, f, g, h, i	4	III: Set up the test to verify the influence of different damage degrees

**Table 3:** Comparison of damage identification results.

Extraction Time	Experiment Number	Condition Type	Accuracy	Average Accuracy of Condition Type
1 min	(1)~(8)	I	58.33%	58.48%
	(9)~(14)	II	65.45%	
	(15)	III	51.67%	
3 min	(1)~(8)	I	88.89%	89.51%
	(9)~(14)	II	91.67%	
	(15)	III	87.96%	
5 min	(1)~(8)	I	90.28%	93.98%
	(9)~(14)	II	97.22%	
	(15)	III	94.44%	
10 min	(1)~(8)	I	100%	100%
	(9)~(14)	II	100%	
	(15)	III	100%	

## Data Availability

The dataset used in this study is from reference [45], which was conducted within the Intelligent Structural Health Monitoring System (ISMO) project, funded by the Multidisciplinary Institute of Digitalisation and Energy (MIDE) research programme at Aalto University, Finland (http://mide.tkk.fi/en/).
